# New Therapies of Neovascular AMD—Beyond Anti-VEGFs

**DOI:** 10.3390/vision2030031

**Published:** 2018-07-30

**Authors:** Praveen Yerramothu

**Affiliations:** School of Optometry and Vision Science, University of New South Wales, Sydney 00098, Australia; prav.yerramothu@unsw.edu.au; Tel.: +61-(2)-93854536

**Keywords:** neovascular AMD, new therapies, anti-VEGFs, AMD signaling

## Abstract

Neovascular age-related macular degeneration (nAMD) is one of the leading causes of blindness among the aging population. The current treatment options for nAMD include intravitreal injections of anti-vascular endothelial growth factor (anti-VEGF). However, standardized frequent administration of anti-VEGF injections only improves vision in approximately 30–40% of nAMD patients. Current therapies targeting nAMD pose a significant risk of retinal fibrosis and geographic atrophy (GA) development in nAMD patients. A need exists to develop new therapies to treat nAMD with effective and long-term anti-angiogenic effects. Recent research on nAMD has identified novel therapeutic targets and angiogenic signaling mechanisms involved in its pathogenesis. For example, tissue factor, human intravenous immune globulin, interferon-β signaling, cyclooxygenase-2 (COX-2) and cytochrome P450 monooxygenase lipid metabolites have been identified as key players in the development of angiogenesis in AMD disease models. Furthermore, novel therapies such as NACHT, LRR and PYD domains containing protein 3 (NLRP3) inflammasome inhibition, inhibitors of integrins and tissue factor are currently being tested at the level of clinical trials to treat nAMD. The aim of this review is to discuss the scope for alternative therapies proposed as anti-VEGFs for the treatment of nAMD.

## 1. Introduction

Age related macular degeneration (AMD) is the most common cause of irreversible blindness among the elderly population [1,2]. Current global prevalence of AMD stands at 170 million and with aging as a major risk factor, it is expected to increase to 288 million by the year 2040 [3]. AMD can be classified into early, intermediate, and advanced forms, depending on the severity of the symptoms [4,5]. Early and intermediate AMD, also referred to as dry AMD (non-exudative), is characterized by accumulation between the retinal pigment epithelium (RPE) and Bruch’s membrane of a yellowish-lipid rich protein content known as drusen leading to functional loss of the retinal photoreceptors [4]. Drusen deposition is considered as the hallmark of AMD [4,5]. The advanced form of AMD is known as geographic atrophy (GA) and is characterized by the loss of RPE and choroid near the macular region leading to the loss of photoreceptors and central vision [4,5]. The severe form of AMD (exudative) presents with growth of abnormal blood vessels from the choroid extending into the avascular RPE and sub-retinal regions. This phenomenon is known as choroidal neo-vascularization (CNV) and the form of AMD with CNV is termed nAMD [4,5]. nAMD accounts for the majority of cases of blindness in AMD patients [1,3].

The formation of neovascularization in AMD is a complex process involving multiple signaling pathways mediated by vascular endothelial growth factor (VEGF), platelet-derived growth factor, fibroblast growth factor, transforming growth factor, the Wnt pathway, the NLRP3 inflammasome, mitogen-activated protein kinase (MAPK) signaling, interleukins and chemokines [6,7,8,9,10]. VEGF-A, a potent pro-angiogenic factor, has been implicated in the pathogenesis of nAMD through CNV [11]. RPE produces VEGF-A via two major pathways: complement activation and oxidative stress [12,13]. Overproduction of VEGF-A leads to the breakdown of the blood-retinal barrier and formation of new blood vessels into the retina. Leakage of blood from these abnormal vessels results in edema and loss of vision if left untreated [9]. Immune cells such as microglia (resident macrophages in the retina), along with chemokines such as CCL2, are known to contribute to CNV and retinal inflammation in AMD pathogenesis [14,15]. Inflammation and its role in AMD has been discussed in previous reviews [10,16]. The focus of the current review is to emphasize novel treatment modalities of nAMD beyond anti-VEGFs—those in clinical trials, as well as some promising candidates in pre-clinical phases of study.

## 2. Current Treatment Modalities of nAMD

### 2.1. Anti-VEGF Injections

Injection of VEGF inhibitors into the vitreous is the current standard for nAMD treatment [17]. However, intravitreal injection of VEGF inhibitors does not provide a ‘cure’ for AMD, but only slows disease progression [18,19]. Agents including ranibizumab, bevacizumab, pegaptanib, and aflibercept have been approved by Food and Drug Administration (FDA) for the treatment of nAMD [18]. Ranibizumab and bevacizumab are humanized monoclonal antibodies that inhibit all isoforms of VEGF-A [18]. Pegaptanib, which binds and inhibits the activation of VEGF-A is a 28 base-pair RNA aptamer and the first anti-VEGF agent approved for use in humans [18,20,21]. Aflibercept is a human recombinant protein that acts as a VEGF decoy receptor, sequestering VEGF [18,22]. Other anti-VEGF agents considered for nAMD treatment include brolucizumab, abicipar pegol, and RG7716, which are currently being tested in phase I, II, and III clinical trials [23,24,25,26,27].

### 2.2. Photodynamic Therapy

Photodynamic therapy (PDT) for nAMD involves intravenous injection of verteporfin, an approved FDA agent [28,29]. Injected verteporfin binds to abnormal blood vessels to exert its anti-angiogenic effects [28,29]. However, treatment with anti-VEGF agents is considered superior, as PDT has been reported to cause damage to the endothelial cells, and results in thrombosis and secondary platelet adhesion [18].

## 3. Rationale for Developing New Therapies

Current treatment strategies for nAMD require repeated, frequent intravitreal injections [18]. Long-term administration of intravitreal anti-VEGF injections is associated with increased risk of developing retinal scarring, and geographic atrophy in nAMD patients two to five years after initiating treatment [30,31]. Furthermore, recent reports from multiple studies suggest that intravitreal injections of anti-VEGF drugs could result in complications such as vitreous and subconjunctival hemorrhage, fluid accumulation under the fovea, increased intra-ocular pressure, endophthalmitis, and ocular inflammation [30,32,33,34,35]. Results from the multi-center SEVEN-UP study show that only one-third of the patients enrolled in the ANCHOR and MARINA trials had an improved visual outcome, leaving the other third with poor outcomes after seven years of ranibizumab therapy [36]. Considering that the current therapies for nAMD are associated with multiple adverse events, there is a clear need to develop novel therapies to treat nAMD.

## 4. New Therapies for nAMD—Thinking beyond Anti-VEGFs

### 4.1. Semaphorin 3F

**Figure 1:**
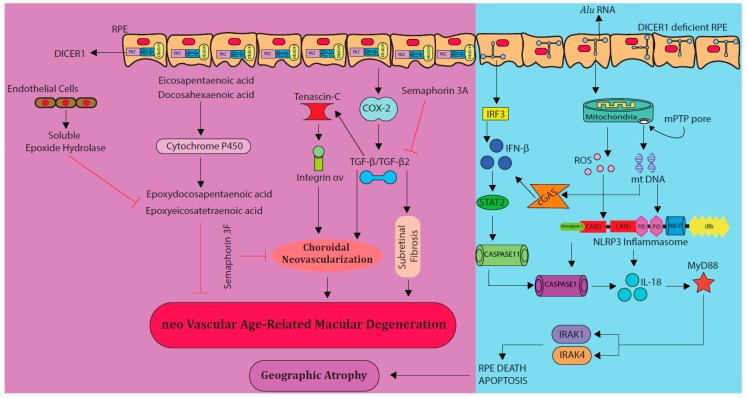
Major signaling pathways involved in the development of nAMD other than vascular endothelial growth factor (VEGF) (purple panel on the left-hand side). Cytochrome P450, cyclooxygenase-2 (COX-2), and Transforming growth factor-β (TGF-β) pathways play critical role in CNV leading to nAMD. On the other hand, semaphorins (3A, 3F) and cytochrome P450 metabolites naturally inhibit the formation of choroidal neovascularization (CNV). DICER 1 enzyme plays a crucial role in Alu RNA breakdown, preventing GA. In DICER 1-deficient RPE (blue panel on the right-hand side), Alu RNA deposits in retinal pigment epithelium (RPE), activating the NLRP3 inflammasome, and cyclic GMP-AMP synthase (cGAS) activates the noncanonical inflammasome, ultimately leading to apoptosis of RPE, and geographic atrophy (GA) development. ROS—reactive oxygen species.

Semaphorins were initially discovered as molecules that contribute to the development of the embryonic nervous system [37]. Semaphorin 3F (Sema3F) is a member of class 3 semaphorin proteins and is expressed in the outer retina under normal conditions and the inner retina during hypoxia [38]. Previously, Sema3F was reported to be protective against subretinal vascularization in mouse models [39]. Sun et al. investigated the anti-angiogenic role of Sema3F in two different mouse models, a very low-density lipid-receptor knockout model of spontaneous subretinal neovascularization where the resultant lesions are comparable to retinal angiomatous proliferations in humans and laser-induced CNV mimicking human nAMD [39]. It was found that intravitreal injection of AAV2. Sema3F effectively inhibited subretinal neovascularization and CNV in both models (Figure 1) [39]. Considering these data, Sema3F could be a potential target around which to design novel therapies for nAMD.

### 4.2. Tissue Factor Inhibition

Tissue factor (TF) is a transmembrane receptor for plasma coagulation factor VII [40,41]. Studies have reported that TF is one of the key mediators in pathological neovascularization and thrombosis [42]. Under normal physiological conditions TF is not expressed by cells, however vascular endothelial cells, monocytes and macrophage express TF in response to inflammation [43]. Increased expression of TF has been observed in the RPE of nAMD patients compared to non-AMD retinas [42,44]. Intravitreal injection of anti-TF monoclonal antibody contributed to the reduction of CNV in a mouse model [45]. With this evidence, TF has been identified as a novel target with which to treat nAMD through the development of hI-con1. hI-con1, a synthetic molecule coupled with factor VII conjugated to the Fc region of an antibody, selectively binds to TF and destroys pathological vessels [46]. hI-con1 is being tested in a multi-center phase II clinical trial, with results pending (ClinicalTrials.gov identifier: NCT02358889) [46].

### 4.3. Targeting the Cytochrome P450 Monooxygenase Pathway

Cytochrome P450 (CYP) is a class of enzymes that can synthesize fatty acid metabolites [47]. CYP monooxygenase is a CYP enzyme that plays a vital role in the metabolism of long-chain polyunsaturated fatty acids (LCPUFAs) into epoxydocosapentaenoic acids (EDPs) and epoxyeicosatetraenoic acids (EEQs), ultimately regulating vascular function (Figure 1) [47]. Previous studies have shown that LCPUFAs derived CYP monooxygenase metabolites; 17,18-EEQ and 19,20-EDP are associated with the regulation of CNV in mouse models (Figure 1) [48]. The intake of diet enriched with eicosapentaenonic acid (EPA) and docosahexaenonic acid (DHA) reduced the severity of nAMD in mice by increasing the plasma levels of EEQs and EDPs [48]. Furthermore, direct treatment of mice with intraperitoneal injections of 17,18-EEQ and 19,20-EDP reduced CNV [48]. CYP2C8 is a potent monooxygenase that converts EPA to 17,18-EEQ and DHA to 19,20 EDP [47]. Overexpression of CYP2C8, 17,18-EEQ, and a diet enriched in 19,20-EDP significantly inhibited CNV in nAMD mice [49]. 17,18-EEQ and 19,20-EDP inhibited CNV by downregulating the expression of cell adhesion molecules, intracellular adhesion molecule-1 (ICAM-1) and E-selectin [49]. ICAM-1 and E-selectin contribute to the formation of CNV as cells expressing these markers were abundantly found in the site of CNV shortly after laser injury [50]. All of this evidence suggests that CYP monooxygenase plays a vital role in inhibiting CNV via LCPUFAs metabolites. In a separate study by Fu et al. oral supplements of ω3 and ω6-LCPUFAs to CNV-induced mice correlated with reduced risk of nAMD development [51]. Oral or dietary ω3, ω6-LCPUFAs, 17,18-EEQ, and 19,20-EDP could serve as a non-invasive treatment modality for nAMD patients [49,51].

Despite the reported protective role of the ω3, ω6-LCPUFAs (DHA, EPA), in reducing nAMD in animal models, results from the Age-Related Eye Disease Study 2 (AREDS2) a multi-center, randomized, double masked phase III human clinical trial, report conflicting results [52]. AREDS2 tested whether the addition of ω3, ω6-LCPUFAs to the original AREDS formulation containing vitamin C, vitamin E, beta-carotene, zinc, and copper would improve the clinical outcome in AMD patients [52]. Results from AREDS2 concluded that the use of ω3, ω6-LCPUFAs did not reduce the risk of AMD progression [52]. On the other hand, a randomized prospective study, Nutritional AMD Treatment 2 (NAT2), has been reported, simultaneous to AREDS2. NAT2 investigated the relationship between ω3, ω6-LCPUFAs in AMD patients [53,54]. NAT2 reported that the incidence and the time-to-occurrence of CNV was not significantly different between the DHA and the placebo group [53,54]. However, a further detailed analysis of NAT2 patient data revealed that individuals with high levels of red blood cell membrane (RBCM) EPA + DHA (good marker for LCPUFAs) are at reduced risk to develop nAMD, suggesting that RBCM EPA + DHA can be a clinically useful biomarker of AMD [54]. Clearly, there are few similarities and differences between the AREDS2 and NAT2 studies (discussed in detailed [25]) in terms of the number of participating patients and the variety of treatment groups that may account for reported differences.

### 4.4. Interleukin-33 Therapy

Interleukin-33 (IL-33), a pro-inflammatory cytokine, is a member of the type-2 IL-1 family [55]. Once activated, IL-33 signals via its receptor, ST2 and the IL-1R accessory protein [56]. In humans, IL-33 is expressed in epithelial cells, endothelial cells and fibroblasts, and in rodents, its expression has been detected in RPE, the inner retina, and the choroid, along with the lymph nodes, spleen and central nervous system (CNS) [55,57,58]. In mouse experimental autoimmune uveitis, systemic administration of IL-33 attenuated the disease severity [58]. Similarly, Theodoropoulou et al. reported a protective role of IL-33 in a laser-induced CNV mouse model [59]. Intravitreal injection of recombinant IL-33 inhibited the development of CNV in mice via inhibition of ST2-expressing fibroblasts and endothelial cells, but did not alter the levels of VEGF [59]. This study discovered a novel mechanism involved in attenuating CNV independent of VEGF signaling, suggesting that recombinant IL-33 therapy could serve as an alternative treatment for nAMD patients who do not respond to conventional anti-VEGF treatments.

### 4.5. Targeted Intraceptor Nanoparticle Therapy

Targeted intraceptor nanoparticle therapy is a three-component system that consists of (1) plasmids expressing *Flt23k* intraceptors, (2) PLGA-biodegradable nanoparticles, and (3) the tripeptide adhesion motif Arg-Gly-Asp (RGD) [60]. Flt23k intraceptors are composed of VEGF-binding domains 2–3 of *Flt*, a high affinity VEGF receptor, while RGD facilitates the selective localization of nanoparticles to CNV after intravenous injection [60]. The *Flt23k* component inhibited CNV, and the RGD component suppressed fibrosis in mice and primates [60]. Although this is an anti-VEGF strategy to inhibit CNV, it has advantages over the conventional treatment for nAMD, (intravitreal injections) that is associated with pain, retinal detachment, and scarring [60]. Targeted intraceptor nanoparticle therapy is administered intravenously and can cross the blood-retinal barrier, which is generally a major obstacle for intravenous delivery. Furthermore, this therapy is nontoxic in vivo, suggesting that this could provide a means for alternative drug delivery route to treat nAMD [60,61].

### 4.6. Targeting MyD88 Pathway and DICER 1

GA is an advanced form of AMD for which there is no current effective treatment [9]. GA can occur over time in patients after repeated administration of anti-VEGFs [62,63,64,65]. Hence, it is important to understand the molecular mechanism involved in the pathogenesis of GA to identify novel therapeutic targets. RPE degeneration leading to loss of photoreceptor function is commonly seen in the patients with GA [9,66]. This RPE degeneration is associated with the accumulation of *Alu* RNA which was previously shown to cause RPE cell death (Figure 1) [66]. *Alu* RNA elements are non-coding genomic sequences that constitute almost 11% of the human genome [67]. They belong to a class of retroelements termed SINEs (short interspersed elements) and are primate-specific [67,68]. Tarallo et al. recently discovered that *Alu* RNA accumulates in GA patients due to a deficiency of the enzyme DICER 1 that functions to cleave *Alu* RNA [8]. The accumulated *Alu* RNA activated the NLRP3 inflammasome (Figure 1) and triggered IL-18-dependent MyD88 signaling in the RPE [8]. The NLRP3 inflammasome is an intracellular NOD-like receptor that operates in innate immunity [69]. Upon activation, it cleaves pro-IL-1β and pro-IL-18 into their biologically active forms via caspase-1 [69]. Pharmacological inhibition of the NLRP3 inflammasome, MyD88, or IL-18 in mouse models and human RPE cell cultures prevented RPE cell death resulting from DICER 1 deficiency [66]. Furthermore, activation of caspase-11 (caspase-4 in humans) in mice has been implicated in the pathogenesis of GA [70]. This activation was mediated by cyclic GMP-AMP synthase (cGAS) leading to IFN-β production and gasdermin D-dependent IL-18 secretion (Figure 1) [70]. Elevated levels of gasdermin D, IFN-β, caspase-4, and cGAS have also been observed in the RPE of human eyes with GA [70]. Discovery of these series of events from DICER 1 deficiency in RPE to cGAS is a breakthrough in understanding the pathogenesis of GA and opens new platforms for novel therapies to treat GA [66,70].

### 4.7. Interferon-β Therapy

Immune cells such as microglia and mononuclear phagocytes play important roles in angiogenesis [71,72]. Microglia, the resident macrophages in the retina, are attracted to the choroid and RPE during CNV [14]. Inhibition of monocyte (precursors for macrophages) migration into the retina reduced CNV in a laser-induced mouse model, suggesting that microglia and monocyte derived macrophages may be pro-angiogenic [71,73]. Targeting the signaling pathways involving macrophage migration could be of therapeutic benefit in neovascular diseases such as AMD [74]. Interferon-beta (IFN-β), via interferon-alpha/beta receptor (IFNAR) signaling, has been identified as a critical pathway in regulating autoimmunity and monocyte/microglia influx in the CNS [75,76]. Luckoff et al. investigated the role of IFN-β and its receptor IFNAR in a laser-induced CNV mouse model and reported that IFN-β and IFNAR play a pivotal role in retinal microglia/macrophage activation and infiltration [77]. IFNAR knock-out mice presented with increased microglia/macrophage activation and CNV [77]. Intraperitoneal injection of recombinant human IFN-β 1a to CNV-induced wild-type mice significantly attenuated CNV formation, vascular leakage, and microglia/macrophage infiltration, suggesting that systemic IFN-β therapy could be a promising treatment option for nAMD patients [77]. Since IFN-β therapy is a well-established treatment option for multiple sclerosis and autoimmune encephalomyelitis, it could have a great potential for treating neovascular diseases such as AMD [78,79,80]. Systemic treatment could reduce the complications resulting from intravitreal injections. However, IFN-β therapy is associated with side-effects such as flu-like symptoms, increased spasticity, menstrual disorders, muscle pain, and headache [81,82]. These-side effects should be taken into consideration in optimizing and designing IFN-β therapies to treat nAMD.

### 4.8. Intravenous Injection of Immune Globulin

Intravenous immune globulin (IVIg) is pooled plasma from thousands of healthy donors with a diverse antibody repertoire and specificity [83]. IVIg has been approved by the FDA for the treatment of primary immunodeficiency diseases [84,85]. The first record of IVIg use dates to the year 1981 for the treatment of thrombocytopenic purpura in children [86]. Bogdanovich et al. reported that human IgG1 is a potent anti-angiogenic factor acting via Fc-mediated signaling through the FcγR1 receptor, a high-affinity receptor for IgG1 [87]. Based on these facts, Yasuma et al. tested the anti-angiogenic properties of IVIg, which is composed of approximately 60% IgG1 in five different humanized mouse models of angiogenesis [88]. Intravenous and intravitreal administration of IVIg in nAMD mice suppressed angiogenesis effectively and attenuated macrophage infiltration, a key factor in promoting angiogenesis [88]. Most importantly, IVIg inhibited neovascularization via the activation of the FcγR1 receptor, a VEGF-independent pathway [88]. As intravenous administration of IVIg effectively suppressed CNV as effectively as intravitreal injections, this could provide an alternative mode of treatment to repeated intravitreal injection of anti-VEGFs in nAMD patients [88].

### 4.9. Inhibitors of Integrins

Integrins are transmembrane proteins that bind to extracellular matrix proteins such as laminin, fibronectin and collagens [89]. Integrins are localized on the surface of RPE and mediate the process of phagocytosis of the outer segment particles of the photoreceptors by RPE [90,91]. Members of the integrin family α5β1, αvβ3, and αvβ5 are expressed during CNV and their antagonists could possibly have a therapeutic role in inhibiting CNV in AMD patients (Figure 1) [92,93,94].

Integrin α5β1 is a fibronectin receptor which is linked to endothelial cell migration and proliferation. ATN-161 is a small peptide inhibitor of Integrin α5β1 [95]. Intravitreal injection of ATN-161 after laser photocoagulation inhibited CNV leakage and neovascularization in rats [95]. Optical coherence tomography and histological examination indicated that treatment with ATN-161 significantly reduced the size of laser-induced lesions in rats [95]. JSM6427 is also an inhibitor of integrin α5β1. Intravitreal injection of JSM6427 significantly attenuated neovascularization in laser-induced CNV in mice [96]. A phase I clinical trial (ClinicalTrials.gov Identifier: NCT00536016) evaluated the pharmacological efficacy and safety of JSM6427 intravitreal injections in 36 patients. The study ended in 2009 with promising results however, to date no further studies have been undertaken to investigate JSM6427.

Volociximab is a monoclonal antibody (Ophthotech Corporation, Princeton, NY, USA) that inhibits the binding of fibronectin to integrin α5β [97]. A phase I clinical trial (ClinicalTrials.gov identifier: NCT00782093) which evaluated the safety of Volociximab as intravitreal injection in combination with ranibizumab, reported positive results [97].

ALG-1001 is a synthetic oligopeptide (Allegro Ophthalmics, San Juan Capistrano, CA, USA) that attenuates α5β1, αvβ3, and αvβ5 integrin mediated blood vessel growth [92]. Phase I and II b clinical trials (ClinicalTrials.gov Identifier: NCT01749891, NCT02348918) reported that ALG-1001 was safe to administer via intravitreal injection, and that it improved the visual acuity of both nAMD and diabetic macular edema patients [92].

Tenascin-C is an extracellular glycoprotein which is mainly expressed in the CNS during developmental stages and its levels are upregulated under inflammatory conditions where it is localized within the CNV membranes of AMD patients (Figure 1) [98,99]. Tenascin-C promotes retinal neovascularization in proliferative diabetic retinopathy patients [100]. Kobayashi et al. reported that tenascin-C co-localizes with integrin αvβ3 in the CNV membranes of AMD patients and laser-induced CNV mice [101]. Furthermore, tenascin-C promoted CNV in mice by binding to integrin αvβ3. Tenascin-C knock out and tenascin-C messenger RNA (mRNA)-silenced (intravitreal injection of siRNA) mice showed a significant reduction in CNV formation, suggesting that tenascin-C-mediated integrin αvβ3 could be a potential target for nAMD treatment [101].

### 4.10. COX-2 Inhibitors

Cyclooxygenases (COXs) are a group of enzymes that are involved in inflammatory immune responses required for the conversion of arachidonic acid to prostaglandins [102]. Out of the three COX isoforms (COX-1, COX-2, and COX-3), COX-2 mediates inflammation and is induced by pathological stimuli [102]. In mice, COX-2 involvement has been implicated in CNV and subretinal fibrosis of the retina (Figure 1). Subretinal fibrosis was associated with the upregulation of transforming growth factor-β2 (TGF-β2) by COX-2 in AMD (Figure 1) [103,104]. Zhang et al. reported that the expression of COX-2 in CNV, and inhibition of COX-2 using NS-398 a COX-2-selective antagonist significantly attenuated CNV and subretinal fibrosis via the inhibition of macrophage infiltration, TGF-β2, and VEGF [105].

### 4.11. Inhibition of CCR3

CCR3 (also known as CD193) is a cell surface chemokine receptor that is expressed by eosinophils, Th2 cells, basophils, and mast cells [106]. CCR3 expression by choroidal endothelial cells was recently reported in CNV membranes excised from nAMD patients [107]. Inhibition of CCR3 using intravitreal injection of anti-CCR3 antibody, a small molecular CCR3 antagonist, or by using CCR3 knockout mice significantly attenuated the formation of CNV in mice [107]. Furthermore, a comparison of CCR3 neutralization versus anti-VEGF treatments in mice reported that CCR3 inhibition was superior to anti-VEGF treatment. [107]. On the contrary, results from a recent study that investigated the broad inhibitory outcome of CCR3 resulted in retinal cell death in laser-induced CNV mice [108]. Intravitreal delivery of CCR3 inhibitor in laser-induced CNV mice led to the apoptosis of Muller cells and reduced the survival of retinal cells surrounding CNV lesions [108]. Inhibition of CCR3 can be considered as a potential target for the treatment of nAMD; however, further studies examining the safety of CCR3 inhibitors and targeting of choroidal endothelial cell CCR3 inhibition might provide greater insight.

## 5. Targeting Other Signaling Pathways with Involvement of VEGF

Several other pathways and novel agents that involve VEGF signaling were recently implicated in the pathogenesis of nAMD. Such mechanisms/agents include but are not limited to the complement pathway, BMP9/ALK1 signaling, erythropoietin signaling, long non-coding RNAs, STAT3 activation, neuropilin 1, platelet activating factor, mTOR signaling, and Yes-associated protein (YAP) inhibitors. Some of these pathways act in parallel (BMP9/ALK1 signaling), downstream (neuropilin 1) or upstream of VEGF signaling (complement pathway, BMP9/ALK1 signaling, platelet-activating factor receptor, STAT3 activation, TGF-β signaling). Since the primary aim of this review is to focus on the pathways independent to VEGF, other signaling pathways that inhibit VEGF directly or indirectly with possible therapeutic benefits in nAMD are not discussed in detail, but are listed in form of table (Table 1), briefly discussing the involvement of key molecules, signaling pathways, and their mechanisms of action.

## 6. Concluding Remarks

The current review highlights a broad range of non-VEGF pathway targets that offer potential new treatments for nAMD. Intravitreal injection of anti-VEGFs has been the standard treatment of nAMD for many years. Despite the success of anti-VEGFs, there is no improvement in vision for one-third of nAMD patients, and the long-term use of anti-VEGF therapy is associated with adverse events such as the development of GA and retinal fibrosis, to name a few. Therefore, a need exists to develop improved strategies that can reduce or eliminate ocular injections and improve clinical outcomes. Recent research has identified many molecular targets other than anti-VEGFs, as well as alternative drug delivery routes, which are currently being tested at the level of clinical trials, offering potential new avenues for treating nAMD. It could also be postulated that employing multiple targeted approaches to treat nAMD could yield better results than single pathway targeting, especially for simultaneous treatment of nAMD and subretinal fibrosis.

## Figures and Tables

**Table 1:** List of potential targets for nAMD treatment partially involving VEGF.

	Signaling/Inhibitor	Key Molecules/Proteins Involved	Findings	Reference
**1**	Complement pathway	C3a, C5a, monocyte chemoattractant protein-1 (MCP-1), VEGF, and MG4 domain, IL-17, γδ T-cells	Antibody-mediated neutralization of C3a, C5a, MG4 domains of β chain, or pharmacological inhibition of their receptors inhibited CNV in mouse nAMD	Jo et al., 2017 [109]; Nozaki et al., 2006 [12]; Tan et al., 2015 [110]; Coughlin et al., 2015 [111]; Robrer et al., 2009 [112]
**2**	BMP9/Alk1 signaling	BMP9, Alk1, VEGF, and VEGFR2	Activating Alk1 signaling inhibited growth of blood vessels in nAMD mouse model	Ntumba et al., 2016 [113]
**3**	Erythropoietin signaling	Erythropoietin, macrophages, CCL2, CXCL10, CCL22, IL-6, and IL-10	Increased erythropoietin signaling is associated with increased CNV in mice	Bretz et al., 2018 [114]
**4**	Long non-coding RNAs	MAPK signaling, Vax2osl, and Vax2os2	326 or 51 long non-coding RNAs that play a role in human nAMD were identified and their dysregulation could provide novel insights into nAMD treatments	Xu et al., 2014 [115]
**5**	Neuropilin 1 (Nrp1)	Nrp1, and VEGF	Reduced CNV was seen in Nrp1 knockout mice	Fernandez-Robredo et al., 2017 [116]
**6**	Platelet-activating factor (PFA)	PFA, PFA-receptor (PFA-R), macrophages, VEGF, MCP-1, and IL-6	WEB2086, a novel PAF-R antagonist, inhibited CNV and experimentally induced subretinal fibrosis in mice	Zhang et al., 2013 [117]
**7**	Nucleoside reverse transcriptase inhibitors (NRTIs)	VEGF-A, and P2X7 receptor	Intravitreal injection of NRTIs, lamivudine, zidovudine, abacavir, and P2X7 antagonist A438079 reduced CNV in mice	Mizutani et al., 2015 [118]
**8**	RG7716 antibody	VEGF, and angiopoietin 2	Phase II clinical trial underway. Phase I results indicated improvement in visual acuity in patients, and that RG7716 was safe	Chakravarthy et al., 2017 [26]
**9**	STAT3 signaling	Monocytes, macrophages, CX3CR1, HLA-DR, STAT3, VEGF, Suppressor of Cytokine Signalling 3	Inhibition of STAT3 activation using LLL12-attenuated CNV in mice and intermediate monocytes (CD14^+^ CD16^+^) are activated in nAMD patients	Chen et al., 2016 [119]
**10**	TGF-β signaling	TGF-β, Smad2/3, VEGF, and TNF-α	Inhibition of TGF-β using a synthetic inhibitor, LY2157299 or Decorin, a natural TGF-β inhibitor significantly inhibited CNV in mice	Wang et al., 2017 [120]
**11**	Yes-associated protein (YAP) signaling	YAP, proliferating cell nuclear antigen (PCNA), CD31, VEGF	YAP small interfering RNA (siRNA) and ranibizumab treatment reduced VEGF and PCNA, reduced endothelial cell proliferation, and CNV formation in mice	Yan et al., 2018 [121]
**12**	Adeno-associated virus-mediated gene therapy with cartilage oligomeric matrix protein angiopoietin-1 (AAV2.COMP-Ang1)	VEGF, and hypoxia-inducible factor (HIF)-α	Subretinal injection of AAV2.COMP-Ang1 reduced VEGF levels and inhibited CNV in mice	Lambert et al., 2016 [122]
**13**	Fenofibric acid (Feno-FA) signaling	Feno-FA, VEGF, TNF-α, ICAM-1, and peroxisome proliferator–activated receptor-alpha (PPARα)	Feno-FA injections in mice suppressed neovascularization	Qiu et al., 2017 [123]
**14**	mTOR signaling	hypoxia-inducible gene *RTP801*, VEGF	A phase II clinical trial reported that the use of siRNA and PF-04523655 in combination with ranibizumab compared to ranibizumab alone improved vision in nAMD patients	Nguyen et al., 2012 [124]
**15**	Connective growth factor (CTGF)	CTGF, and ERK signaling	RXI-109, an inhibitor of CTGF, is designed to reduce retinal fibrosis in nAMD patients. Phase I clinical trial is currently underway	Kothary et al., 2010 [125]; ClinicalTrials.gov identifier: NCT02599064
